# Analysis of the correlation between lipotoxicity and pituitary-thyroid axis hormone levels in men and male rats

**DOI:** 10.18632/oncotarget.10045

**Published:** 2016-06-14

**Authors:** Jianmei Yang, Xiaoming Zhou, Xu Zhang, Jianting Hu, Ling Gao, Yongfeng Song, Chunxiao Yu, Shanshan Shao, Zhongshang Yuan, Yan Sun, Huili Yan, Guimei Li, Jiajun Zhao

**Affiliations:** ^1^ Department of Endocrinology, Shandong Provincial Hospital Affiliated to Shandong University, Jinan, Shandong, China; ^2^ Institute of Endocrinology and Metabolism, Shandong Academy of Clinical Medicine, Jinan, Shandong, China; ^3^ Shandong Clinical Medical Center of Endocrinology and Metabolism, Jinan, Shandong, China; ^4^ Shandong Academy of Pharmaceutical Sciences, Jinan, Shandong, China; ^5^ Scientific Center, Shandong Provincial Hospital Affiliated to Shandong University, Jinan, Shandong, China; ^6^ Department of Epidemiology and Biostatistics, School of Public Health, Shandong University, Jinan, Shandong, China; ^7^ Department of Pediatric Endocrinology, Shandong Provincial Hospital Affiliated to Shandong University, Jinan, Shandong, China

**Keywords:** lipotoxicity, high-fat lard diet, hypertriglyceridemia, anterior pituitary hormones, pituitary-thyroid axis, Pathology Section

## Abstract

Lipotoxicity seriously harms human health, but it is unclear whether lipotoxicity is detrimental to the pituitary. We investigated the correlation between serum triglyceride and pituitary axis hormone levels in epidemiological and animal studies. In the epidemiological study, serum thyroid-stimulating hormone (TSH), follicle-stimulating hormone (FSH) and luteinizing hormone (LH) levels were greater in male patients with isolated hypertriglyceridemia than in controls, whereas adrenocorticotropin (ACTH) levels were lower in the patients with hypertriglyceridemia. Pituitary hormone levels correlated with triglyceride levels, even after adjustment for potential confounders. In the animal study, male rats were fed a high-fat or control diet for 28 weeks. As the duration of high-fat feeding increased, the serum and pituitary triglyceride concentrations increased. At early times, the high-fat diet elevated serum TSH and triiodothyronine. At later times, much higher serum TSH levels coupled with reduced thyroxine were observed in the high-fat group. Serum levels of pituitary-gonadal and pituitary-adrenal axis hormones were not affected by the diet. The mRNA and protein expression of *Tshβ* were greater in the high-fat group than in the control group, whereas expression of *Fshβ*, *Lhβ* and *Acth* had no difference between the groups. Overall, serum triglyceride levels were associated with pituitary-thyroid axis hormone levels.

## INTRODUCTION

Hypertriglyceridemia, characterized by elevated triglycerides (TGs) in the blood [1], is a vital risk factor threatening human health [2]. High circulating TG levels promote the accumulation of neutral lipids as TGs in non-adipose tissues, contributing to chronic cellular dysfunction and injury [3–5], which is referred to as lipotoxicity. In recent years, it has become well known that lipotoxicity seriously and extensively harms human health by promoting the pathogenesis of metabolic diseases [6–8]. In 2014, we reported that lipotoxicity induced abnormal functioning of the rat thyroid [5]. Pituitary controls the serum thyroid hormone levels, but to date, no research has explored whether the pituitary is another organ damaged by lipotoxicity.

The pituitary gland, a crucial neuroendocrine organ, synthesizes and secretes different kinds of pituitary hormones [9]. It also participates in controlling multiple endocrine organs. There are three classic, mature pituitary axes: the pituitary-thyroid, pituitary-gonadal and pituitary-adrenal axes. The pituitary-thyroid axis hormones, including thyroid-stimulating hormone (TSH) and the thyroid hormones, control the function of the thyroid and are essential for metabolic homeostasis [10]. For example, the pituitary can increase or reduce the TSH level according to the feedback of the thyroid hormones [11]. Pituitary-gonadal axis hormones, including follicle-stimulating hormone (FSH), luteinizing hormone (LH) and testosterone (T), control the gonadal function and are essential for reproduction [12]. Finally, pituitary-adrenal axis hormones, including adrenocorticotropin (ACTH) and cortisol (COR), control the function of the adrenal gland and are essential for stress responses [13]. Abnormal hormone levels can lead to metabolic disorder and other diseases [14, 15]. Thus, maintaining normal pituitary axis hormone levels is very important for human health.

In the present study, we first conducted an epidemiological analysis to assess the correlation between serum TG and pituitary axis hormone levels. Second, to verify this clinical phenomenon and investigate whether long-term TG overload correlates with pituitary-thyroid axis function, we established a hypertriglyceridemic rat model and evaluated not only its serologic changes, but also its mRNA and protein level changes. Our findings suggested that pituitary-thyroid axis hormone levels correlate with lipotoxicity, providing further evidence of the widespread deleterious effects of hypertriglyceridemia on the body.

## RESULTS

### Hypertriglyceridemia was connected with elevated serum TSH, FSH and LH and reduced ACTH in the epidemiological study population

To determine whether pituitary axis hormone levels correlated with serum TG levels, we conducted an epidemiological study. The selected subjects were assigned into two groups: healthy control group (*n* = 60) or isolated hypertriglyceridemia group (*n* = 30). Table 1 displays the study subjects' general characteristics and serum pituitary axis hormone levels. No significant difference was discovered between the two groups about age, systolic blood pressure (SBP), diastolic blood pressure (DBP), fasting plasma glucose (FPG), Hemoglobin A1c, total cholesterol (TC) or low-density lipoprotein cholesterol (LDL-C). Compared with the control, serum TG, body mass index (BMI), waist circumference and waist-to-hip ratio (WHR) were significantly increased in the isolated hypertriglyceridemia group. Regarding the pituitary-thyroid axis, serum TSH was significantly (approximately 1.5-fold) higher, while serum free thyroxine (FT4, also known as tetraiodothyronine) was lower, in the human subjects with isolated hypertriglyceridemia than in the control subjects, although free triiodothyronine (FT3) did not differ between the two groups. Regarding the pituitary-gonadal axis, the levels of FSH and LH were 25.9% and 24.4% greater, respectively, in the human subjects with isolated hypertriglyceridemia than in control subjects, while the level of serum T was decreased in the isolated hypertriglyceridemia group. Regarding the pituitary-adrenal axis, ACTH was reduced by 32.7% and COR was elevated in the human subjects with isolated hypertriglyceridemia relative to controls. These results demonstrated an association of hypertriglyceridemia with pituitary axis hormone levels.

**Table 1:** Clinical or laboratory characteristics and the pituitary hormone levels of study participants

	Healthy control (n = 60)	Isolated hypertriglyceridemia (n = 30)	*p* value
Age (yr), mean (SD)	51.07 (3.97)	51.43 (4.75)	0.717
SBP (mmHg), mean (SD)	123.08 (9.69)	121.79 (12.94)	0.597
DBP (mmHg), mean (SD)	76.18 (6.45)	76.60 (8.35)	0.792
FPG (mmol/L), mean (SD)	5.45 (0.29)	5.54 (0.45)	0.372
HbA1c (%), mean (SD)	5.60 (0.30)	5.60 (0.24)	0.916
BMI (kg/m^2^), mean (SD)	22.95 (2.48)	26.43 (3.28)	<0.001
WC (cm), mean (SD)	84.68 (6.16)	89.76 (9.86)	0.014
WHR, mean (SD)	0.88 (0.05)	0.93 (0.06)	<0.001
TC (mmol/L), mean (SD)	4.07 (0.89)	4.32 (0.83)	0.207
TG (mmol/L), mean (SD)	1.00 (0.32)	2.90 (1.42)	NA
LDL-C (mmol/L), mean (SD)	2.44 (0.65)	2.17 (0.67)	0.070
Pituitary-thyroid axis			
TSH (mIU/L),median (IQR)	1.43 (1.16)	2.17 (1.47)	0.001
FT3 (pmol/L), mean (SD)	5.18 (0.58)	5.06 (0.75)	0.419
FT4 (pmol/L), mean (SD)	17.32 (2.20)	16.29 (1.82)	0.030
Pituitary-gonadal axis			
FSH (mIU/mL), mean (SD)	6.16 (1.84)	7.76 (3.21)	0.015
LH (mIU/mL), mean (SD)	4.96 (1.35)	6.17 (1.90)	0.003
T (ng/mL), mean (SD)	6.47 (2.03)	4.62 (1.44)	<0.001
Pituitary-adrenal axis			
ACTH (pg/mL), mean (SD)	13.41 (6.76)	9.03 (3.14)	<0.001
COR (nmol/L), mean (SD)	405.15 (153.34)	480.83 (130.21)	0.023

The groups were compared using the Student's *t*-tests or the Mann-Whitney test. *P* values less than 0.05 were considered statistically significant.

Abbreviations: NA, not applicable; SBP, systolic blood pressure; DBP, diastolic blood pressure; FPG, fasting plasma glucose; HbA1c, hemoglobinA1c; BMI, body mass index; WC, waist circumference; WHR, waist hip ratio; TC, total cholesterol; TG, triglycerides; LDL-C, low-density lipoprotein cholesterol; TSH, thyroid-stimulating hormone; FT3, free triiodothyronine; FT4, free thyroxine; FSH, follicle-stimulating hormone; LH, luteinizing hormone; T, testosterone; ACTH, adrenocorticotropin; COR, cortisol.

As shown in Table 2, among all patients, serum TG significantly positively correlated with TSH, FSH and LH. In contrast, TG levels negatively correlated with ACTH levels. Interestingly, the correlations still kept significant after ajustment for age, BMI, FPG and SBP, and even after an additional adjustment for the corresponding target gland hormones (TSH was adjusted for FT3 and FT4, while both FSH and LH were adjusted for T, and ACTH was adjusted for COR).

**Table 2:** Pearson correlation analysis of serum TG and pituitary hormones

	Log TSH	FSH	LH	ACTH
TG unadjusted	r = 0.260	r = 0.280	r = 0.298	r = −0.317
	*p* = 0.013	*p* = 0.007	*p* = 0.004	*p* = 0.002
TG adjusted ^a^	r = 0.254	r = 0.290	r = 0.344	r = −0.326
	*p* = 0.018	*p* = 0.006	*p* = 0.001	*p* = 0.002
TG adjusted ^b^	r = 0.249	r = 0.250	r = 0.349	r = −0.262
	*p* = 0.022	*p* = 0.020	*p* = 0.001	*p* = 0.015

Abbreviations: TG, Triglyceride; TSH, thyroid-stimulating hormone; FSH, follicle-stimulating hormone; LH, luteinizing hormone; ACTH, adrenocorticotropin; FT3, free triiodothyronine; FT4, free thyroxine; T, testosterone; COR, cortisol.

TSH was transformed to its natural logarithm to optimize the models. A value of *p* < 0.05 was considered statistically significant.

a Adjusted for Age, BMI, FPG and SBP.

b Adjusted for Age, BMI, FPG, SBP and corresponding target gland hormones (LogTSH was adjusted for FT3 and FT4; both FSH and LH were adjusted for T; ACTH was adjusted for COR).

Multiple regression analysis appeared similar results (Table 3). The independent correlates of the serum pituitary hormones were evaluated after age, BMI, FPG, SBP and the corresponding target gland hormone levels were taken into account. TG levels were positively associated with TSH, FSH and LH levels and negatively associated with ACTH levels.

**Table 3:** Multiple regression analysis of serum TG and pituitary hormones in the study

Variables	TG (mmol/L)				
	B	SE	95% CI of B	Beta	*p* value
LogTSH (mIU/L)	0.045	0.018	0.010, 0.081	0.260	0.013
FSH (mIU/mL)	0.561	0.205	0.154, 0.967	0.280	0.007
LH (mIU/mL)	0.394	0.135	0.127, 0.662	0.298	0.004
ACTH (pg/mL)	−1.240	0.493	−2.219, −0.260	−0.251	0.014

Data are B (unstandardized coefficients), SE (corresponding standard error), 95% CI of B, Beta (standardized coefficients) and *p* value

for linear regression analysis of TG.

Abbreviations: TG, Triglyceride; TSH, thyroid-stimulating hormone; FSH, follicle-stimulating hormone; LH, luteinizing hormone;

ACTH, adrenocorticotropin; FT3, free triiodothyronine; FT4, free thyroxine; T, testosterone; COR, cortisol.

Values shown were adjusted for Age, BMI, FPG, SBP and corresponding target gland hormones (LogTSH was adjusted for FT3 and FT4; both FSH and LH were adjusted for T; ACTH was adjusted for COR).

TSH was transformed to its natural logarithm to optimize the models. A value of *p* < 0.05 was considered statistically significant.

We further used a generalized linear model to analyze the correlation between serum TG and pituitary hormone levels. There was a consistent increase in TSH, FSH and LH and a decrease in ACTH with increasing TG. Table 4 displays the obvious linear trend between TG and TSH (*p* = 0.001), FSH (*p* = 0.013), LH (*p* = 0.004) and ACTH (*p* < 0.001). Thus, subjects with lower serum TG had slightly lower TSH, FSH and LH and higher ACTH than subjects with higher serum TG. The present results fully clarified a positive correlation between TG and TSH, FSH or LH, and a negative correlation between TG and ACTH, independent of confounding factors.

**Table 4:** Estimated marginal means of serum TSH, FSH, LH and ACTH levels according to triglyceride categories

	LogTSH			FSH			LH			ACTH		
	Mean	SE	95% CI	Mean	SE	95% CI	Mean	SE	95% CI	Mean	SE	95% CI
Triglyceride quartile												
Quartile 1 (<0.97 mmol/L)	0.147	0.037	0.070-0.224	6.151	0.448	5.227-7.075	4.932	0.292	4.328-5.536	14.751	1.492	11.670-17.831
Quartile 2 (0.97-1.36 mmol/L)	0.205	0.048	0.106-0.304	6.235	0.303	5.613-6.858	5.047	0.198	4.640-5.453	13.744	1.229	11.219-16.269
Quartile 3 (1.37-1.99 mmol/L)	0.294	0.044	0.200-0.387	6.728	0.564	5.539-7.917	5.432	0.463	4.455-6.408	9.443	0.703	7.961-10.925
Quartile 4 (>1.99 mmol/L)	0.342	0.039	0.259-0.424	7.944	0.769	6.334-9.554	6.276	0.437	5.362-7.190	8.290	0.640	6.951-9.629
Linear coefficient	0.260			0.280			0.298			−0.317		
*p* value for linear trend	0.001			0.013			0.004			< 0.001		

Data are estimated marginal mean (mean), corresponding standard error (SE) and 95% confidence interval (CI).

Abbreviations: TSH, thyroid-stimulating hormone; FSH, follicle-stimulating hormone; LH, luteinizing hormone; ACTH, adrenocorticotropin.

Values shown were adjusted for Age, BMI, FPG, SBP and corresponding target gland hormones.

TSH was transformed to its natural logarithm to optimize the models. A value of *p* < 0.05 was considered statistically significant.

### A high-fat (HF) diet elevated rats serum TG levels

**Figure 1:**
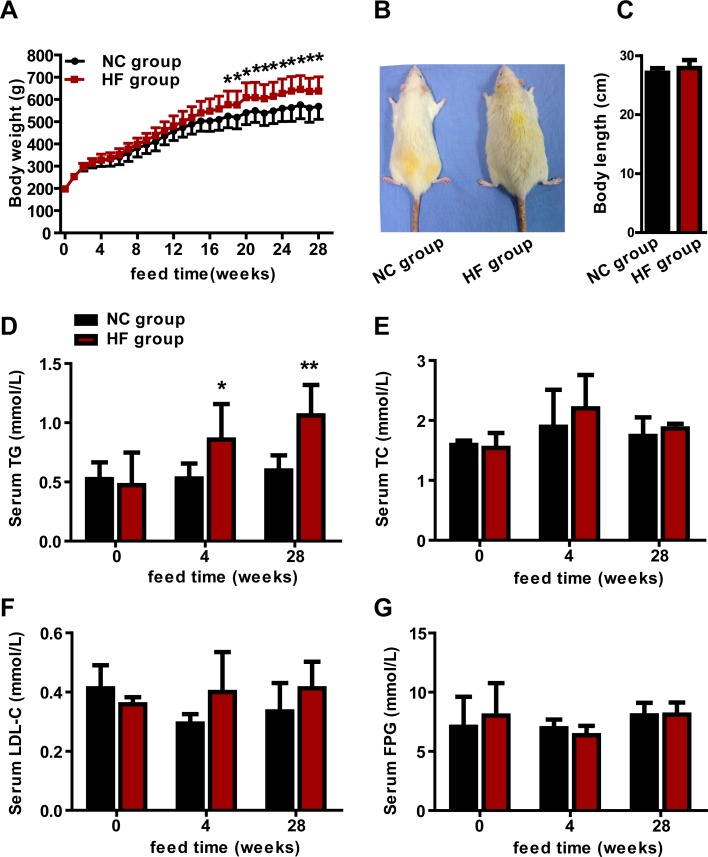
The effect of a HF diet on rat body weight, body length and serum lipid profile Rats were fed a NC diet or a HF diet. **A.** The body weights of the rats were monitored weekly. **B.** Representative gross morphology of rats at 28 weeks. **C.** Body lengths of rats at 28 weeks. The serum TG **D.**, TC **E.**, LDL-C **F.** and FPG **G.** levels of the rats were assayed at 0, 4 and 28 weeks. The data are expressed as the mean ± SD (*n* = 20 per group). * *p* < 0.05, ** *p* < 0.01 *versus* the corresponding NC group.

We found that serum TG levels were obviously correlated with pituitary axis hormone concentations in our epidemiological study. To verify this clinical phenomenon and to explore the progressive effects of lipotoxicity on the pituitary, we established a diet-induced hypertriglyceridemic rat model. All rats were weighed weekly. No obvious differences were found between the two groups about the body weights before 17 weeks. However, compared with the normal control (NC) group, body weights were increased in the HF group from the 18th to 28th week (*p* < 0.05, Figure 1A). We did not find the obvious difference of body length between the two groups at the end of 28 weeks (Figures 1B and 1C).

To survey the effects of the HF diet on serum lipid profile and FPG, we examined serum TG, TC, LDL-C and FPG levels. Compared with the NC group, serum TG had increased by 61.8% at 4-week (*p* < 0.05) and by 77.9% at 28-week (*p* < 0.01) in the HF group (Figure 1D), while no obvious difference in TC, LDL-C or FPG between the two groups at any time point (Figure 1E, 1F and 1G). These results indicated that a HF diet increased serum TG levels, and that we successfully set up a rat model of hypertriglyceridemia.

### A HF diet increased rat pituitary TG and free fatty acid contents

**Figure 2:**
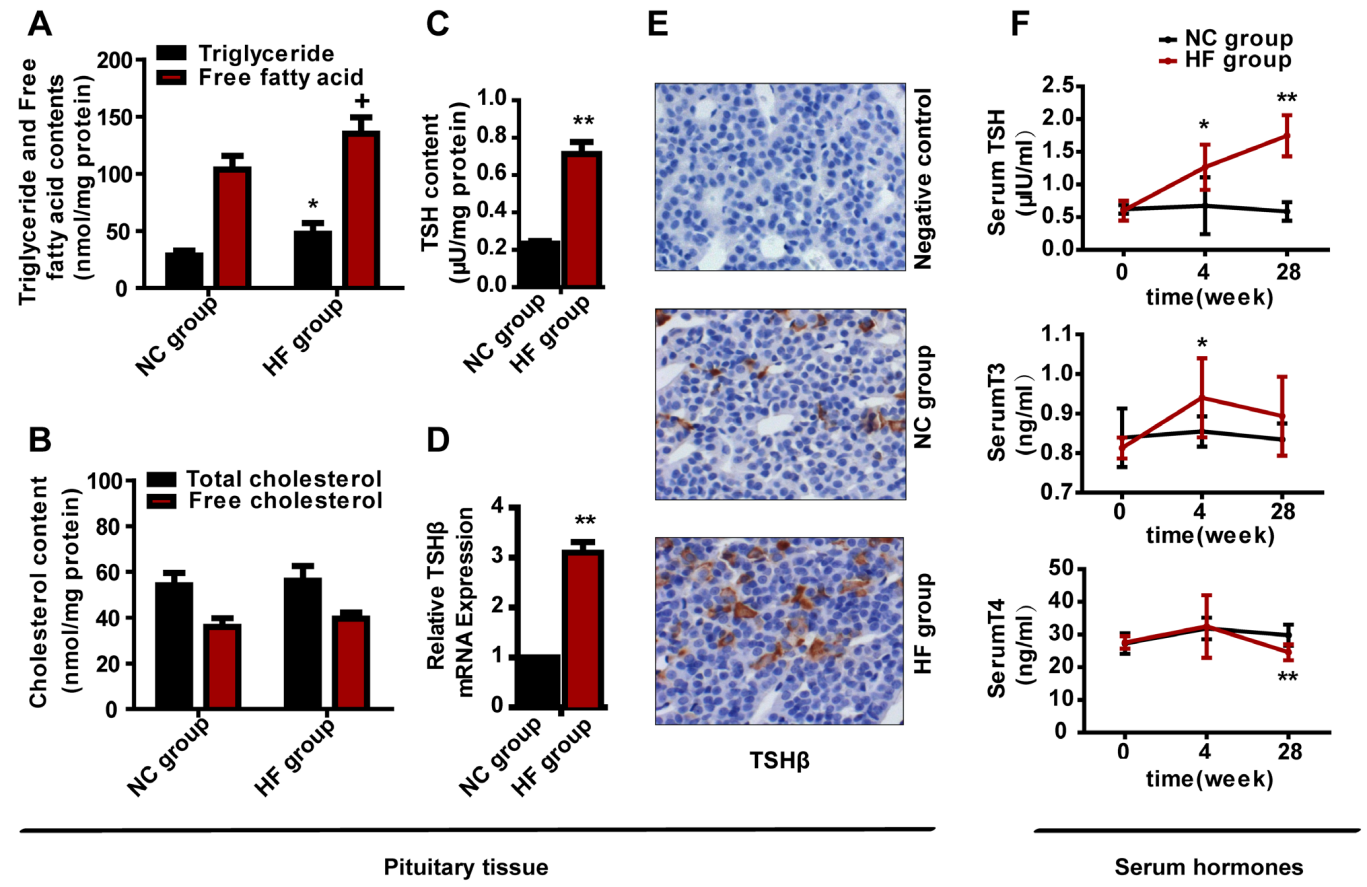
The effect of a HF diet on rat pituitary-thyroid axis hormone levels Rats were fed a NC diet or a HF diet for 28 weeks. **A.** Rat pituitary TG and free fatty acid contents were assayed and corrected against the corresponding total protein content. **B.** Rat pituitary TC and free cholesterol contents were assayed and corrected against the corresponding total protein content. **C.** TSH content in each pituitary. The total pituitary content of TSH was measured by ELISA and corrected against the total protein content. **D.** *Tshβ* gene expression. **E.** Representative images of TSHβ immunohistochemical staining are shown (magnification, × 200). All positive immunostaining is indicated by brown in the cytoplasm. **F.** Serum TSH, T3 and T4 levels at 0, 4 or 28 weeks. The data are expressed as the mean ± SD (*n* = 20 per group). + *p* < 0.05, * *p* < 0.05, ** *p* < 0.01 *versus* the corresponding NC group.

To observe the alteration of lipid content in the pituitary after HF diet feeding, we measured the TG, free fatty acid, TC and free cholesterol contents of the pituitary tissues at 28 weeks. Surprisingly, as shown in Figure 2A, the pituitary TG and free fatty acid contents of the HF group were approximately 1.7- and 1.3-fold (both *p* < 0.05) higher than those of the NC group, whereas the total and free cholesterol contents had no obvious difference between the two groups (Figure 2B). Our results suggested that a HF diet increased the TG and free fatty acid contents in the pituitary.

### A HF diet increased the mRNA and protein expression of *Tshβ*

To survey whether a HF diet was connected with the protein expression of TSH, we first used an enzyme-linked immunosorbent assay (ELISA) to quantify the TSH content of extract from each pituitary. Compared with the NC group, the TSH content was increased about 207.3% in the HF group (p < 0.01) (Figure 2C).

TSH includes α-subunit and β-subunit, and the β-subunit is responsible for TSH specificity and endows biological activity on TSH [9, 16, 17]. Thus, as we explored the effect of the HF diet on the TSH level, we directly evaluated the transcription of β-subunit. Compared with the NC group, the mRNA expression of *Tshβ* in pituitary gland was elevated by 3.1-fold in the HF group (*p* < 0.01) (Figure 2D).

We also performed immunohistochemical analysis of TSHβ protein in the anterior pituitaries of rats from the HF diet and control groups. Scattered and inhomogeneous distribution of the staining granules was evident in the cytoplasm of the anterior pituitary. We found the similar results with the expression of *TSHβ* mRNA (Figure 2E).

### A HF diet altered pituitary-thyroid axis hormone levels

To investigate whether pituitary-thyroid axis dysfunction occurred following HF diet feeding, we measured serum TSH, T3 and T4 levels. No significant difference in serum TSH, T3 and T4 levels between the two groups at 0 weeks, but differed following HF diet intake. At 4 weeks, serum TSH level was 1.9-fold greater in the HF group than in the NC group (*p* < 0.05), and the serum T3 concentration also increased in the HF group (*p* < 0.05), while the T4 concentration remained normal. At 28 weeks, the TSH level was 3-fold (*p* < 0.01) greater in the HF group than in the NC group, while the T4 concentration was reduced in the HF group (*p* < 0.01) and the T3 level was normal (Figure 2F). Thus, the HF diet caused pituitary-thyroid axis dysfunction, and the change in serum TSH was similar to the changes in *Tshβ* mRNA and protein expression.

### A HF diet did not alter pituitary-gonadal or pituitary-adrenal axis hormone levels

**Figure 3:**
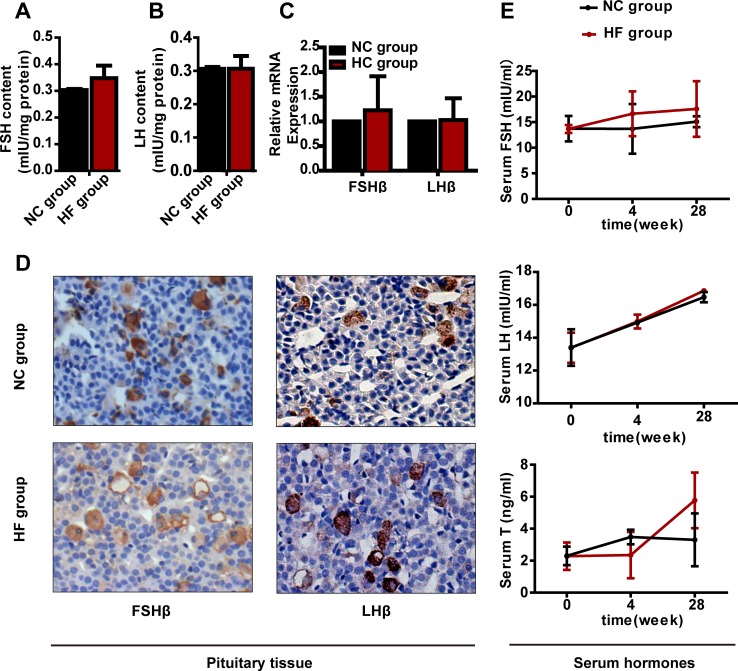
The effect of a HF diet on rat pituitary-gonadal axis hormone levels Rats were fed a NC diet or a HF diet for 28 weeks. **A.** and **B.** The total pituitary FSH and LH contents were measured by ELISA and corrected against the corresponding total protein content. **C.** Real-time PCR analysis of *Fshβ* and *Lhβ* in the pituitary. **D.** Representative images of FSHβ and LHβ immunohistochemical staining are shown (magnification, × 200). All positive immunostaining is indicated by brown in the cytoplasm. **E.** Serum FSH, LH and T levels at 0, 4 or 28 weeks. The data are expressed as the mean ± SD (*n* = 20 per group). * *p* < 0.05, ** *p* < 0.01 *versus* the corresponding NC group.

To investigate whether pituitary-gonadal or pituitary-adrenal axis dysfunction happened following HF diet feeding, we measured the corresponding hormone levels. The FSH and LH contents, *Fshβ* or *Lhβ* mRNA or protein expression in the pituitary did not differ obviously between the two groups (Figures 3A, 3B, 3C and 3D). In addition, serum FSH, LH and T had not changed significantly after either 4 or 28 weeks of HF diet treatment (Figure 3E).

**Figure 4:**
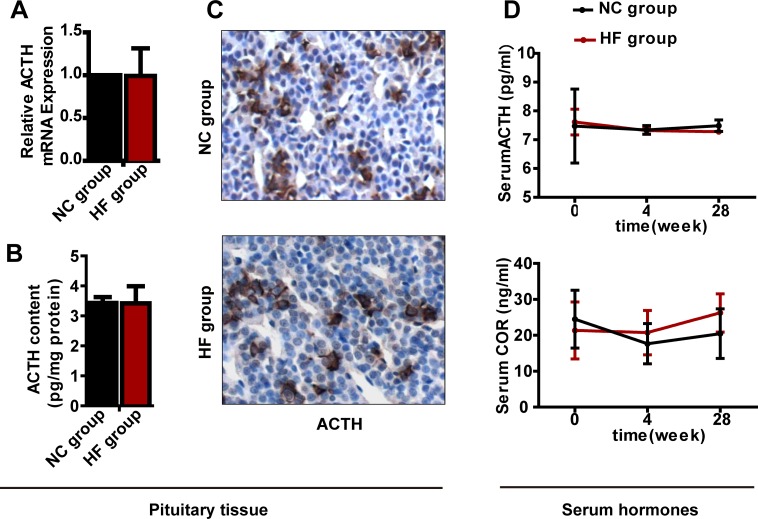
The effect of a HF diet on rat pituitary-adrenal axis hormone levels Rats were fed a NC diet or a HF diet for 28 weeks. **A.** *Acth* gene expression. **B.** The total pituitary content of ACTH was measured by ELISA and corrected against the total protein content. **C.** Representative images of ACTH immunohistochemical staining are shown (magnification, × 200). All positive immunostaining is indicated by brown in the cytoplasm. **D.** Serum ACTH and COR levels at 0, 4 or 28 weeks. The data are expressed as the mean ± SD (*n* = 20 per group). * *p* < 0.05, ** *p* < 0.01 *versus* the corresponding NC group.

For the pituitary-adrenal axis, similar to the above results for the pituitary-gonadal axis, neither mRNA nor protein expression of *Acth* differed significantly between the two groups, and serum ACTH and COR levels had not changed significantly after either 4 or 28 weeks of HF diet treatment (Figure 4).

## DISCUSSION

Our study provides strong evidence that there is a significant correlation between lipotoxicity and pituitary axis, especialy pituitary-thyroid axis, hormone levels. The present study indicates that the pituitary may be another target organ damaged by lipotoxicity, providing further evidence of the extensive and serious harm of lipotoxicity to the entire body.

Lard is widely consumed food that is rich in saturated fatty acids, and is frequently used to study the harm of HF diet [5, 10, 18, 19]. We only added lard, no cholesterol, to standard rodent chow in this study. 0-, 4- and 28-week HF diet studies were conducted to mimic baseline, short-term and long-term exposure, respectively. Serum TG levels increased significantly in rats fed the HF diet under these conditions, in accordance with a report by Louwe *et al*. [20].

Similar to the serum TG, the pituitary TG content also increased in rats fed a HF diet. Yin *et al*. [21] reported that a HF/high-sucrose diet elevated lipid accumulation in non-adipose tissues such as the heart, liver and pancreas. Additionally, a HF diet promoted fat depositing in the liver and caused liver disease in mice [22] and rats [23]. Thus, a HF diet may induce TG deposit in non-adipose tissues. Our results demonstrated that a HF diet also increased pituitary TG content.

A HF diet increased serum TSH and T3 in the early period (4 weeks) in the present study, increased serum TSH but did not change the thyroid hormone levels at 12 weeks in our previous study [5], and increased TSH markedly but reduced serum T4 in the late period (28 weeks) in the present study. Based on these observations, it appears the altered serum TSH level was mainly due to a change in the pituitary gland in the early period. In our animal study, if the elevated TSH levels because of feedback effects of T3 and T4, the T3 and T4 levels would have decreased, but actually serum T3 was increased at 4 weeks. These results illustrate that a HF diet may hyperactivate the pituitary-thyroid axis. However, in the late period, although TSH was elevated in rats, we had no way to exclude the feedback effects from T3 and T4. A damaging effect on the thyroid gland may also have contributed to the elevated serum TSH, but the pituitary was still harmed, as well. We therefore think that direct overstimulation of the pituitary gland caused by lipotoxicity may occur on the one hand, while an indirect toxic effect on the thyroid may occur on the other. This may partly explain our observed effects in the late period.

In our epidemiological study, for the pituitary-gonadal axis, significant positive correlations were still identified between serum TG and FSH or LH after adjustment for T. For the pituitary-adrenal axis, there was a negative correlation between serum TG and ACTH, even after adjustment for COR. This suggests serum TG levels are associated with the levels of these pituitary hormones, independent of their target gland hormones. Mai *et al*. [24] also found that hypertriglyceridemia was associated with ACTH levels, but the authors did not adjust for COR levels. In our animal study, but pituitary-gonadal and pituitary-adrenal axis hormones were unaffected by the HF diet. The reason for the difference between humans and rats unclear, though: Dunkel *et al*. [25] reported that hypertriglyceridemia progressively reduced basal ACTH levels in horses. Given that the effect of lipotoxicity on hormone levels is progressive, the duration of the HF diet may be not enough to affect these hormone concentrations. Species differences may also contribute to the inconsistency of the results. In addition, our epidemiological investigation was a cross-sectional study, so the results may reflect long-term, chronic stimulation.

Cai *et al*. [26, 27] reported that a HF diet induced hypothalamic dysfunction by stimulating inflammatory pathways. Mitochondrial dysfunction [28, 29], oxidative stress [30] and endoplasmic reticulum (ER) stress [31, 32] can atypically trigger inflammatory changes. Zhang *et al*. reported that ER stress in the hypothalamus could be induced by a HF diet [33]. Although there is no direct evidence, the abovementioned studies raise the possibility that inflammation, ER stress, intracellular oxidative stress and mitochondrial dysfunction also participate in pituitary dysfunction.

Our study has several potential limitations. First, we did not measure serum hypothalamic hormones levels, but this does not prevent our conclusions, since we could still observe the changes in pituitary axis function, as shown in a study by Li *et al*. [34]. Second, we did not measure serum fatty acid levels, which also reflect lipotoxicity, because we noted that the serum TG concentration has often been used as an indicator of the severity of lipotoxicity in clinical investigations [35, 36]. Third, although we identified correlations between TG and pituitary function, we did not conduct experiments *in vitro* in pituitary cell lines, so we did not observe direct effects of TG on pituitary function.

In summary, we found a correlation between lipotoxicity and pituitary-thyroid axis hormone levels. Our finding indicates the potential contribute to the disturbances of pituitary-thyroid axis function described in hypertriglyceridemia. It also provides further evidence of the widespread deleterious effects of hypertriglyceridemia in the body.

## MATERIALS AND METHODS

### Part 1: Epidemiological study

#### Subjects

This cross-sectional epidemiological investigation was performed in Ningyang area (Taian, Shandong, China) between June and November 2011. There was 11000 participants in this investigation. All participants were more than 40 years old and lived there at least 5 years. The study was performed in compliance with the Declaration of Helsinki.

The exclusion criteria include: (1) Missing vital data (such as age, sex or serum lipid profile); (2) Female; (3) < 45 or ≥ 60 years; (4) Hypothalamic or pituitary diseases; (5) Diabetes mellitus or hypertension; (6) Malignant tumor, serious liver or renal disorders; (7) Taking medicines that affect serum lipids or pituitary function (such as anti-lipemic agents, thyroid hormones, estrogens, androgens) in the previous three months [37]; (8) Hypercholesterolemia. Then, the remaining participants were stratified according to age, FPG, HbA1c, SBP and DBP, respectively. Finally, 90 men were selected and studied. The specific selection process was shown in the Supplementary Figure 1.

#### Anthropometric measurements and laboratory methods

Weight (kg), height (m), waist circumference (cm) and hip circumference (cm), respectively, were measured. BMI was calculated as the weight divided by the square of height (kg/m^2^). WHR was the ratio of waist circumference and hip circumference. Blood pressure was measured in the sitting position after a 10-min rest.

Blood samples were obtained from all the subjects from 8:00 to 10:00 after a minimum 10-h overnight fast [9, 38, 39]. Serum TSH, FT3, FT4, FSH, LH, T, ACTH and COR levels were determined by chemiluminescent methods (Roche, Basel, Switzerland). The lipid profile and FPG levels were measured using biochemistry analyzer (Olympus, Japan). Hemoglobin A1c was determined by Hemoglobin Testing System (Bio-Rad, USA). Laboratory reference ranges were as follows: TSH: 0.27-4.2 mIU/L, FT3: 3.1-6.8 pmol/L, FT4: 12-22 pmol/L, FSH: 1.5-12.4 mIU/mL, LH: 1.7-8.6 mIU/mL, T: 2.8-8.0 ng/mL, ACTH: 7.2-63.3 pg/mL and COR: 171-536 nmol/L. Hypertriglyceridemia was defined as TG ≥ 1.70 mmol/L (150 mg/dL), and hypercholesterolemia was defined as TC ≥ 5.18 mmol/L (200 mg/dL) and/or LDL cholesterol ≥ 3.37 mmol/L (130 mg/dL) [40].

### Part 2: Animal study

#### Animals and experimental design

To obtain similar serum lipid features to those in part 1, we purchased 40 male Sprague-Dawley rats (body weight 170-190 g; 6 weeks old) from Vital River Laboratory Animal Technology Co. Ltd. (Beijing, China). All rats were kept on a 12/12 hour light/dark cycle at 22°C with unlimited access to water and food. The rats were divided into two groups at random and feed for 28 weeks: (1) the NC diet group (*n* = 20): 100% standard chow (3.4 kcal/g) or (2) the HF diet group (*n* = 20): 85% standard chow supplemented with 15% lard (4.1 kcal/g) [5]. The animals received humane care according to the guidelines of the Animal Care and Use Committee of Shandong Provincial Hospital.

After a 0-, 4- or 28-week treatment, fasting blood samples of all rats were collected from 8:00 to 10:00. Sera were used for the measurement of lipid profile, FPG, TSH, T3, T4, FSH, LH, T, ACTH and COR levels. All rats were sacrificed after 28 weeks feeding. The pituitaries were quickly excised and properly collected. Certain pituitaries were flash-frozen in liquid nitrogen for the assessments of TG, TC, free cholesterol and free fatty acids contents, TSH, FSH, LH and ACTH concentrations, *Tshβ, Fshβ, Lhβ* and *Acth* gene expression. Certain pituitaries were fixed in 4% paraformaldehyde for immunohistochemistry.

### Serum parameter analysis

According to the manufacturer's instructions, serum pituitary hormones and corresponding target gland hormone levels were measured with ELISA kits (Cusabio, Wuhan, China). Serum lipid and FPG levels were measured using an automatic biochemistry analyzer (Olympus, Japan).

### Pituitary lipid concentration assays

According to the manufacturer's instructions, the TG, TC and free cholesterol contents in the pituitary were extracted and measured with kits (Applygen, Beijing, China), and free fatty acids content were determined with a kit (BioVision, CA, USA) [9].

### ELISAs

Using ELISA kits in accordance with the manufacturer's instructions (BIOSAMITE, Shanghai, China) to measure TSH, FSH, LH and ACTH contents in the pituitary. The concentrations in the pituitary were directly measured without dilution. The total protein of each pituitary was measured by the Bradford protein assay kit (Bio-Rad, california, USA). The TSH, FSH, LH or ACTH value for each sample was divided by the corresponding total protein content.

### Quantitative RT-PCR

Quantitative RT-PCR was carried out in accordance with a method previously described [9]. In brief, total RNA was extracted from pituitary with the RNeasy Kit (Qiagen, Canada). One microgram of total RNA was used for cDNA synthesis with the iScript cDNA Synthesis Kit (Takara, Japan). Quantitative RT-PCR reactions were performed in ABI PRISM 7500 Sequence Detection System (Applied Biosystems, Foster City, CA). *β-actin* was used as an internal satandard for normalization. *Tshβ, Fshβ, Lhβ* and *Acth* gene expressions were measured, and the relative expression fold change was calculated by using the 2^−ΔΔCt^ method [41]. Primer sequences of targets are shown in Table 5.

**Table 5:** Quantitative Real-Time PCR Primer Sequences

GENE	FORWARD	REVERSE	GENBANK ACCESSION NUMBER
TSH β	GAC CAT CAA CAC CAC CAT CTG	GGG TAG GAG AAA TAA GGA GCA AC	NM_013116
FSH β	GTC TGC TGC CAT AGC TGT GAA	CAT ACT TTC TGG GTG TTT GGT CTA	NC_005102.3
LH β	ACT GTC CTA GCA TGG TTC	ACA GGA AAG GAG ACT ATG G	NM_012858
ACTH	GCC CTC CTG CTT CAG ACC T	GGC TGT TCA TCT CCG TTG C	NM_139326.2
β-actin	CTA AGG CCA ACC GTG AAA AGA	CCA GAG GCA TAC AGG GAC AAC	NM_031144.3

### Immunohistochemistry

Pituitary tissues were fixed with formaldehyde and embedded in paraffin wax. Sectioned tissue (4 μm) were immunohistochemically stained with TSHβ antibody (Santa, sc-7813, 1:200), FSHβ antibody (Santa, sc-7797, 1:200), LHβ antibody (Abcam, ab150378, 1:1200) and ACTH antibody (Abcam, ab74976, 1:3000), meanwhile, the primary antibody was replaced with normal serum IgG at a similar dilution as a negative control [9, 42]. The antibody reactivity was measured with a kit of Histostain-SP, diaminobenzidine was used for visualising, and then the nuclei was counterstained through hematoxylin. Positive stainings were a brownish-yellow colour.

### Statistical analysis

Values are expressed as the mean ± standard deviation (SD) or median (interquartile range). All data were analyzed using SPSS 18.0 (Chicago, IL, USA). Student's t-test or the Mann-Whitney U test was used to compare groups. Relationships between serum TG and TSH, FSH, LH or ACTH were assessed through Pearson's correlation, partial correlation, multivariate linear regression and generalized linear model analyses. *p* < 0.05 was a statistical significance.

## SUPPLEMENTARY MATERIAL


