# Supplementary material for: Analysis of the correlation between lipotoxicity and pituitary-thyroid axis hormone levels in men and male rats
Source: Oncotarget. 2016 Jun 14;7(26):39332–44. doi: 10.18632/oncotarget.10045 (PMC5129936; doi:10.18632/oncotarget.10045)
Supplement: Supplementary file 1 [file oncotarget-07-39332-s001.pdf]

# Analysis of the correlation between lipotoxicity and pituitary-thyroid axis hormone levels in men and male rats

**Supplementary Material**

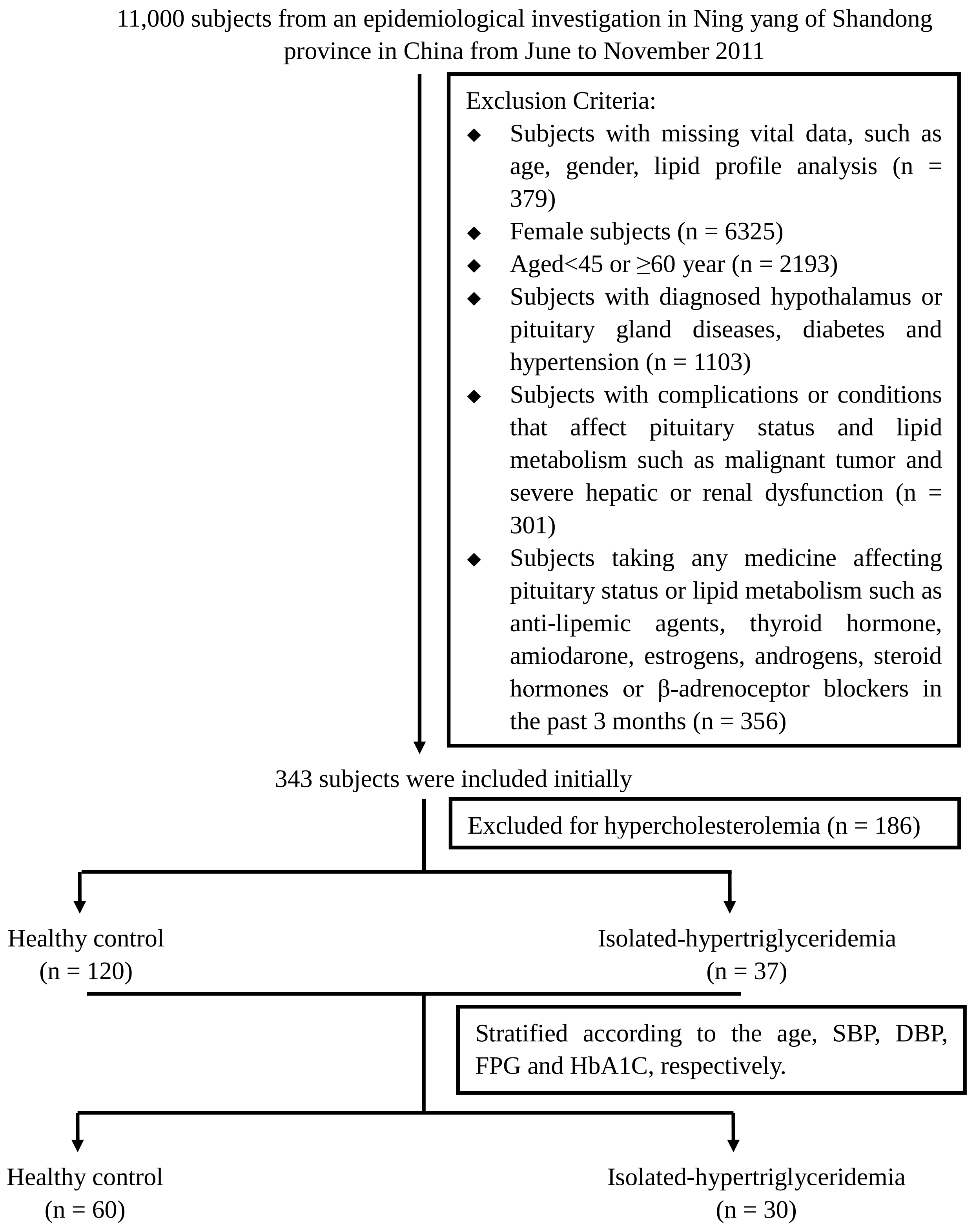

**Fig. S1.** Flowchart of the stratified sampling procedure. A total of 11,000 participants' data were obtained in this study. From the total of 10,621 persons (4296 men and 6325 women) for whom complete data were available, 4296 men were selected. In the next stages, 2193 men aged <45 or ≥ 60 year were excluded and 2103 men aged 45-59 year were included. In the 4$^{th}$ stage, we further excluded 1760 men for hypothalamus diseases, pituitary gland diseases, intake of drugs that influence serum lipids or pituitary function within the past 3 months, malignant tumors, serious liver or renal dysfunctions, diabetes mellitus and hypertension, then excluded 186 subjects for hypercholesterolemia. The final stage of the sampling process was stratified by age, blood glucose and blood pressure status. In addition, control participants and those with isolated hypertriglyceridemia were selected for inclusion in the study. In the end, 90 men were included in the final analysis.
